# Long-Term Follow-Up of Refractory Large Macular Hole with Autologous Neurosensory Retinal Free Flap Transplantation

**DOI:** 10.1155/2022/1717366

**Published:** 2022-05-09

**Authors:** Po-Yen Lee, Yo-Chen Chang, Pei-Kang Liu, Tzu-En Kao, Horng-Jiun Wu, Kuo-Jen Chen, Kwou-Yeung Wu, Kai-Chun Cheng, Wen-Chuan Wu

**Affiliations:** ^1^Department of Ophthalmology, Kaohsiung Medical University Hospital, Kaohsiung 80708, Taiwan; ^2^Department of Ophthalmology, Kaohsiung Municipal Ta-Tung Hospital, Kaohsiung Medical University, Kaohsiung 80145, Taiwan; ^3^Department of Ophthalmology, School of Medicine, Kaohsiung Medical University, Kaohsiung 80708, Taiwan; ^4^Department of Ophthalmology, Park One International Hospital, Kaohsiung 81357, Taiwan; ^5^Department of Ophthalmology, Kaohsiung Municipal Siaogang Hospital, Kaohsiung Medical University, Kaohsiung 81267, Taiwan

## Abstract

**Purpose:**

To evaluate the long-term anatomic and functional outcomes of autologous neurosensory retinal free flap transplantation (ART) for patients with refractory large macular hole (MH).

**Design:**

Retrospective interventional case series.

**Methods:**

We reviewed 9 patients who underwent ART for their refractory large MH. In this extended follow-up study, postoperative assessment including spectral-domain optical coherence tomography and best-corrected visual acuity (BCVA) were recorded at 12, 15, 18, 21, and 24 months after surgery.

**Results:**

The macular hole of all patients appeared successfully closed during the whole follow-up period. The mean logMAR BCVA improved from 1.61 ± 0.44 (preoperative) to 0.72 ± 0.30 (12 months after surgery) (*p* < 0.001). Thereafter, the mean BCVA remained stable at each follow-up. At the mean 16.0 ± 0.8 months postoperatively, inner retinal cystic changes were observed in 4 eyes (44.4%), but these did not significantly affect vision.

**Conclusion:**

ART is a good alternative technique for closing large refractory macular holes. Although inner retinal cystic changes were observed in 4 eyes (44.4%), this phenomenon did not significantly affect visual acuity. It provides long-term good anatomical and functional results, especially in cases where insufficient ILM or lens capsule are left.

## 1. Introduction

Kelly and Wendel first described the surgical rationale to manage macular holes (MH) in 1991 [1]. Thereafter, methods to repair this pathology have been continuously refined. In 1997, Eckardt et al. reported that internal limiting membrane (ILM) removal is effective to prevent the recurrence of MH [2]. Nowadays, vitreous surgery combined with ILM peeling has become the standard treatment for MH [3–7], with the closure rate of MH to be as high as 90% [8, 9]. However, for patients with large MH, the risk of surgery failure might be increased, and the MH closure rate is reduced from 40% to 80% by using the ILM peeling technique [10, 11]. Therefore, the ILM flap technique should be considered as the primary treatment for larger MH and high myopic MH, and the MH closure rate could be as high as 98% [12, 13]. Nevertheless, refractory larger MH and high myopic MH usually require another surgical treatment including revitrectomy with extended ILM peeling [14], autologous free ILM flap transplantation [15], or transplantation of lens capsule [16], but the surgical success rate is usually reduced [17]. In 2019, “autologous neurosensory retinal free flap transplantation” was first proposed by Grewal et al. It provides a surgical technique when no sufficient ILM is left to repair the refractory MH [18, 19]. According to the authors, the retinal flap is flattened by perfluoro-n-octane heavy liquid (PFC). If small amounts of PFC are occasionally left in the vitreous cavity, a further operation for PFC removal may be required. In order to simplify and improve the surgical procedure, we adopted Viscoat (Alcon, Fort Worth, TX) or whole blood-assisted autologous neurosensory retinal free flap transplantation (ART) with gas or silicone oil tamponade for refractory large MH [20, 21]. In short-term follow-up of 12 months, a 90% closure rate and significant improvement of visual function were achieved [21]. Although the short-term effect is very impressive, we do not know the long-term outcome of this procedure. Therefore, the aim of this study is to describe the long-term anatomic and functional outcome of ART for patients with refractory large macular hole.

## 2. Patients and Methods

### 2.1. Study Design

The present study was a retrospective and interventional case series. It has been evaluated by the Institutional Review Board of Kaohsiung Medical University Hospital and deemed not to require ethical approval. The study was performed in accordance with the Helsinki Declaration and the International Conference on Harmonization. This study included individuals who had undergone at least two unsuccessful ILM surgeries including ILM peeling, extended ILM peeling, or autologous free ILM flap transplantation for MH between July 2016 and November 2017.

### 2.2. Surgical Technique

All surgeries were performed by the corresponding author (W.C.W.) at the Department of Ophthalmology, Kaohsiung Medical University Hospital. The detailed surgical procedure is described in our previous publication [16]. In brief, each patient underwent standard 25-gauge, 3-port PPV (Constellation; Alcon), applied endolaser photocoagulation to outline the retinal free flap located superior to the arcade, and harvested a neurosensory retinal free flap which was approximately 1.5–2 times the diameter of MH. While demarcating the flap, blood draw from patient's antecubital vein was performed. The selection of adhesives is random. Then, the infusion was closed temporarily to avoid turbulent flow, and the free flap was placed on the surface of the MH with assistance of a drop of the patient's whole blood or a small amount (approximately 0.1 mL) of Viscoat. After the flap was manipulated into a proper position inside the hole, 0.2-0.3 mL of Viscoat was then injected gently to cover the MH, and a fluid-air exchange was then performed. At the end of the surgery, the air was replaced with silicone oil. Six months after the operation, each patient received removal of silicone oil.

### 2.3. Preoperative and Postoperative Examinations

The preoperative and postoperative examinations included best-corrected visual acuity (BCVA) measured by Snellen chart, intraocular pressure, fundus examination by fundus photography, indirect binocular ophthalmoscopy, and OCT imaging using spectral-domain optical coherence tomography (SD-OCT, Heidelberg Retina Angiograph 2; Heidelberg Engineering, Heidelberg, Germany).

In the present study, postoperative assessments were planned at 12, 15, 18, 21, and 24 months postoperatively. Best-corrected visual acuity using a Snellen chart was converted to the logarithm of minimum angle of resolution (logMAR) for analytical purposes.

### 2.4. Statistical Analysis

Statistical analyses were performed using Student's *t*-test and Fisher's exact test by IBM SPSS Statistics 24.0. *P* values of less than 0.05 were considered statistically significant.

## 3. Results

A total of 9 patients (3 males and 6 females; age range 40–77 years; mean age 63.6 ± 11.4 years) with refractory large macular hole who underwent successful autologous neurosensory retinal free flap transplantation participated in this extended observational study. The clinical characteristics and demographics of the 9 patients are given in Table 1. The preoperative mean diameter of the MH was 1437.6 ± 586.4 *μ*m.

### 3.1. Anatomic Results

After 24 months of long-term follow-up, the transplanted flap tissue was still adhered tightly to the surrounding retinal tissue in all 9 eyes (100%). However, inner retinal cystic changes were observed on SD-OCT in 4 eyes (44.4%) at the mean 16.0 ± 0.8 months postoperatively (range, 15–17 months) (Table 1).

### 3.2. Functional Results

**Figure 1:**
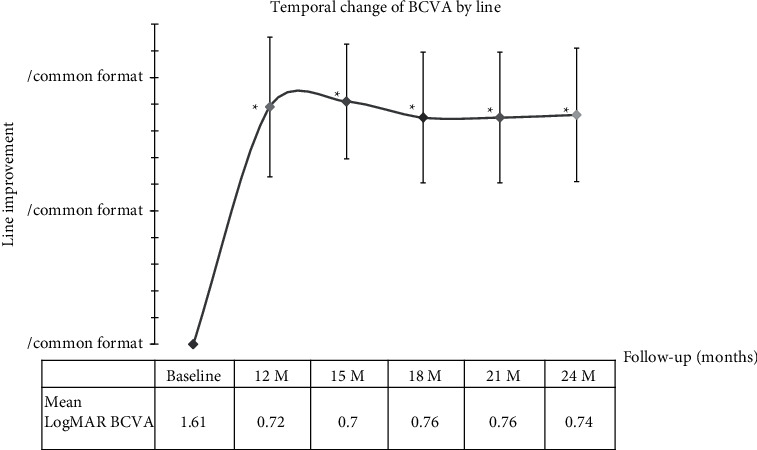
Postoperative temporal change of BCVA by line and mean BCVA. At 12 months after surgery, the logMAR BCVA improved to 0.72 ± 0.30 and the mean line improvement was 8.9 ± 2.6 lines from baseline (*p* < 0.001). The vision remained constant after that. The mean line improvement from baseline was 9.1 ± 2.1 lines at 15 months following surgery (*p* < 0.001). The mean logMAR BCVA improved by 8.7 ± 2.5 lines from baseline to 0.74 ± 0.30 at 24 months (*p* < 0.001).

At baseline, the mean logMAR BCVA was 1.61 ± 0.44. At 12 months, the mean logMAR BCVA improved by 8.9 ± 2.6 lines to 0.72 ± 0.30 (*p* < 0.001). Thereafter, the mean BCVA remained stable at each follow-up. At 24 months, the mean BCVA was 0.74 ± 0.30 logMAR which was not statistically different to the mean BCVA at 12 months (*p*=0.346).  Figure 1 shows the preoperative and postoperative visual changes of these 9 patients.

### 3.3. Functional Differences between the Eyes with or without Macular Cystic Change

During the follow-up period, inner retinal cystic changes were observed on SD-OCT in 4 eyes (44.4%) at the mean 16.0 ± 0.8 months postoperatively. In order to understand whether cystic change will affect vision or not, we adopted “mean line change” to compare the functional differences between the eyes with or without macular cystic change. The “mean line change” was defined as logMAR BCVA at 24 months postoperatively compared with the best postoperative logMAR BCVA of each patient. For the eyes without macular cystic change, the mean line change was −0.4 ± 0.55 lines. For the eyes with macular cystic change, the mean line change was −0.75 ± 0.50 lines. The functional difference between these two groups was not statistically significant (*p*=0.356) (Table 2). Furthermore, we compared preoperative parameters between these two groups including number of previous surgeries (*p*=0.292), size of macular hole (*p*=0.724), logMAR BCVA at baseline (*p*=0.107), preoperative cystic change at the edge (*p*=1), and type of adhesive (*p*=1). The differences of parameters between these two groups were statistically insignificant (Table 3).

There were no postoperative problems including flap displacement, epiretinal membrane, endophthalmitis, or retinal detachment during the follow-up period.

### 3.4. Case Presentation

**Figure 2:**
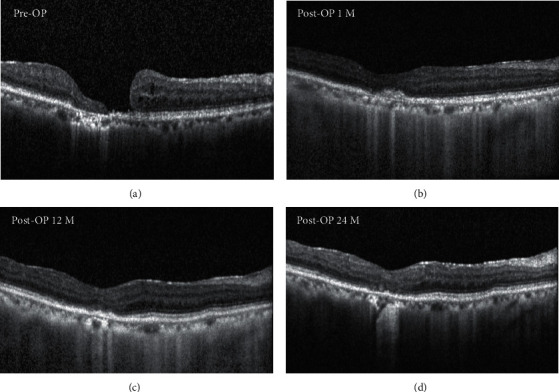
OCT findings after autologous retinal transplantation (ART) of case 1. (a) OCT scan obtained before surgery showing a macular hole with a diameter of 811 *μ*m. The BCVA was 20/200. (b) OCT obtained at postoperative 4 weeks after ART showing early integration of the flap and some glial tissue seen in the outer retina. The BCVA was 20/150. (c) OCT scan obtained at postoperative 12 months showing further reconstitution of the ELM and EZ bands and a decrease of glial tissue. The BCVA was 20/100. (d) OCT scan obtained at postoperative 24 months showing properly positioned in situ of the flap. The BCVA was maintained at 20/100.

**Figure 3:**
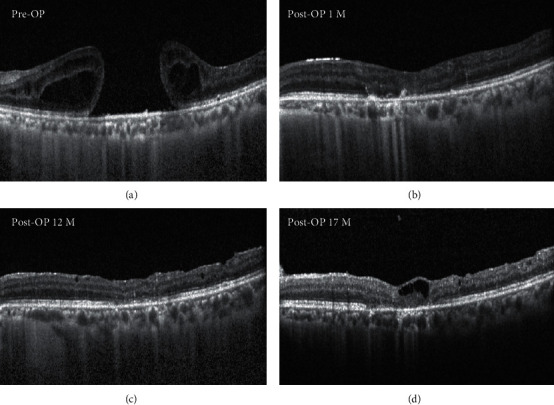
OCT findings after autologous retinal transplantation (ART) of case 5. (a) Baseline OCT showing a large macular hole with a diameter of 2209 *μ*m. The BCVA was 20/2000. (b) One-month follow-up OCT of the same patient. It showed retinal flap integration with the edges of the macular hole. (c) 12-month follow-up OCT. It showed further reconstitution of the ELM and EZ bands. The BCVA was 20/100. (d) Seventeen months postoperation, the inner retinal cystic change was seen on OCT, but BCVA was 20/133.

Figure 2 shows the clinical outcome of an autologous neurosensory retinal free flap transplantation for large MH in a patient. During the 24-month postoperative follow-up, SD-OCT pictures reveal the consistent closure of MH.  Figure 3 shows the clinical outcome of an autologous neurosensory retinal free flap transplantation for a patient with large MH. During the postoperative follow-up, SD-OCT images reveal the closure of MH. However, 17 months postoperation, the inner retinal cystic changes were observed on SD-OCT.

## 4. Discussion

Since Kelly and Wendel first described the surgical rationale to manage MH in 1991 [1], the surgical technique for the repair of MH has undergone a stepwise evolution [2–7]. Generally speaking, the success rate of MH surgery can be as high as 90% [8, 9]. However, compared with idiopathic MHs, the postoperative closure rate and functional outcome of ILM peeing for large MHs are relatively low and reoperations are usually needed [14]. Therefore, the ILM flap technique should be considered as the primary treatment for larger MH, and high myopic MH and the MH closure rate could be as high as 98% [12, 13]. Several surgical techniques, including extended ILM peeling, autologous free ILM flap transplantation, or lens capsular flap transplantation, have been reported for large or refractory MH repair [14–16]. All these methods facilitate the closure of most MHs. However, for patients who still have a persistent MH even after multiple surgeries, a free ILM flap and capsular flap may not be available. Therefore, neurosensory retinal free flap transplantation becomes a reasonable and feasible method for the repair of a refractory MH.

In our previous study, we enrolled 10 patients who had undergone at least two ILM surgeries for MH, but were unsuccessful. For these 10 patients, we used an autologous neurosensory retinal free flap with silicone oil tamponade, and MH closure was accomplished in 9 of them (90%) at a 12-month follow-up [21]. In order to understand the long-term results and complications of this surgical method, we conducted this extended study.

From our present study, in the anatomical result, the transplanted flap tissue was still adhered tightly to the surrounding retinal tissue in all 9 eyes (100%) after 24 months of long-term follow-up. However, by SD-OCT exam, cystoid macular edema (CME) like inner retinal cystic changes was observed in 4 eyes (44.4%), usually at an average of 16 months postoperatively. Although the retinal flap of these four patients had a cystic change, it did not significantly affect vision. The CME like inner retinal cystic changes is not unusual after retinal free flap transplantation. Grewal et al. observed inner retinal cystic change in 7 of 41 eyes in their group, but this did not affect vision, either. To the best of our knowledge, since ART is still a relatively new surgical technique, there is currently no effective way to avoid cystic change after surgery.

Although the exact mechanism remains unknown, the possible explanation of this inner retinal cystic change may be due to insufficient blood supply to the graft retina. In our series, most patients underwent multiple previous surgeries with wide ILM peeling, the underlying and nearby circulation of the chronic unclosed MH might be insufficient to support the physiological function of the retinal free flap, and therefore, splitting of the retinal flap occurred. The second possible explanation is foveolization of the retinal free flap. After the retinal flap was transplanted to MH, in order to restore the structure of the macula, the original stratification within the flap may begin to change, from the original thicker 10 layers gradually thinning, resulting in CME like inner retinal cystic changes.

In summary, we present encouraging long-term surgical outcomes of autologous neurosensory retinal free flap transplantation for refractory large MH. Overall persistent high anatomic success and stable visual function were achieved. During the extended follow-up, 40% of eyes developed CME like inner retinal cystic changes but these did not significantly affect vision. However, our study is limited by its retrospective nature, lack of standardized imaging, and lack of controls. Further exploration is necessary to directly compare this technique with other techniques.

## Figures and Tables

**Table 1:** Pre and postoperative demographics.

Eyes	Age	Sex	Primary disease	No. of OPs for MH	Size of MH (*μ*m)	Pre-OP cystic change at the edge	Adhesives	MH status post-OP	Flap cystic change	Time to cystic change	BCVA	
											12 m	24 m
1	75	F	MH	2	811	Yes	Viscoat	Closed	No	N/A	20/100	20/100
2	69	M	ERM	2	1401	Yes	Blood	Closed	Yes	15	20/1000	20/1000
3	67	F	MH	2	970	No	Viscoat	Closed	Yes	16	20/50	20/60
4	77	F	MH	2	915	No	Blood	Closed	No	N/A	20/133	20/100
5	40	M	MH	2	2206	Yes	Viscoat	Closed	Yes	17	20/100	20/133
6	65	M	RRD	3	1513	Yes	Blood	Closed	Yes	16	20/200	20/250
7	56	F	MH	2	2250	Yes	Blood	Closed	No	N/A	20/50	20/50
8	56	F	MH	2	884	No	Viscoat	Closed	No	N/A	20/50	20/50
9	68	F	MH	2	1988	Yes	Viscoat	Closed	No	N/A	20/133	20/133

BCVA, best-corrected visual acuity; ERM, epiretinal membrane; F, female; MH, macular hole; M, male; Mo, months postoperative; OPs, operations; RRD, rhegmatgenous retinal detachment.

**Table 2:** Functional change between the eyes with or without cystic change.

Status of retinal flap	No. of eyes	Mean line change
Cystic change (−)	5	−0.4 ± 0.55
Cystic change (+)	4	−0.75 ± 0.50
*P* value		0.356

“Mean line change” was defined as “logMAR BCVA of 24 months postoperatively” compared with best postoperative logMAR BCVA.

**Table 3:** Preoperative parameters between the eyes with or without macular cystic change.

	Cystic change (−)	Cystic change (+)	*P* value
No. of OP	2.3 ± 0.4	2.0 ± 0	0.292
Size of MH (*μ*m)	1369.6 ± 691.4	1615.6 ± 512.3	0.724
LogMAR BCVA at baseline	1.4 ± 0.3	1.9 ± 0.5	0.107
Pre-OP cystic change (+) at the edge	3 (50%)	3 (50%)	1
Pre-OP cystic change (−) at the edge	2 (67%)	1 (33%)	
Viscoat	3 (60%)	2 (40%)	1
Blood	2 (50%)	2 (50%)	

BCVA, best-corrected visual acuity; logMAR, logarithm of the minimum angle of resolution; MH, macular hole; No., number; Pre-OP, preoperative.

## Data Availability

The data used to support the findings of this study are available from the corresponding author upon request.

## References

[1] Kelly N. E., Wendel R. T. (1991). Vitreous surgery for idiopathic macular holes: results of a pilot study. *Retina*.

[2] Eckardt C., Eckardt U., Groos S., Luciano L., Reale E. (1997). Removal of the internal limiting membrane in macular holes. clinical and morphological findings. *Der Ophthalmologe*.

[3] Park D. W., Sipperley J. O., Sneed S. R., Dugel P. U., Jacobsen J. (1999). Macular hole surgery with internal-limiting membrane peeling and intravitreous air. *Ophthalmology*.

[4] Lois N., Burr J., Norrie J. (2011). Internal limiting membrane peeling versus no peeling for idiopathic full-thickness macular hole: a pragmatic randomized controlled trial. *Investigative Ophthalmology & Visual Science*.

[5] Fabian I. D., Moisseiev J. (2011). Sutureless vitrectomy: evolution and current practices. *British Journal of Ophthalmology*.

[6] Spiteri Cornish K., Lois N., Scott N. (2013). Vitrectomy with internal limiting membrane (ILM) peeling versus vitrectomy with no peeling for idiopathic full-thickness macular hole (FTMH). *Cochrane Database of Systematic Reviews*.

[7] Spiteri Cornish K., Lois N., Scott N. W. (2014). Vitrectomy with internal limiting membrane peeling versus no peeling for idiopathic full-thickness macular hole. *Ophthalmology*.

[8] Scott I. U., Moraczewski A. L., Smiddy W. E., Flynn H. W., Feuer W. J. (2003). Long-term anatomic and visual acuity outcomes after initial anatomic success with macular hole surgery. *American Journal of Ophthalmology*.

[9] Ando F., Sasano K., Ohba N N., Hirose H., Yasui O. (2004). Anatomic and visual outcomes after indocyanine green-assisted peeling of the retinal internal limiting membrane in idiopathic macular hole surgery. *American Journal of Ophthalmology*.

[10] Kusuhara S., Teraoka Escaño M. F., Fujii S. (2004). Prediction of postoperative visual outcome based on hole configuration by optical coherence tomography in eyes with idiopathic macular holes. *American Journal of Ophthalmology*.

[11] Chhablani J., Khodani M., Hussein A. (2015). Role of macular hole angle in macular hole closure. *British Journal of Ophthalmology*.

[12] Sasaki H., Shiono A., Kogo J. (2017). Inverted internal limiting membrane flap technique as a useful procedure for macular hole- associated retinal detachment in highly myopic eyes. *Eye*.

[13] Maier M., Bohnacker S., Klein J. (2019). Vitrectomy and iOCT-assisted inverted ILM flap technique in patients with full thickness macular holes. *Der Ophthalmologe*.

[14] Sabti K. A., Kumar N., Azad R. V. (2009). Extended internal limiting membrane peeling in the management of unusually large macular holes. *Ophthalmic Surgery Lasers and Imaging Retina*.

[15] Tam A. L. C., Yan P., Gan N. Y., Lam W. C. (2018). The current surgical management of large, recurrent, or persistent macular holes. *Retina*.

[16] Chen S. N., Yang C. M. (2016). Lens capsular flap transplantation in the management of refractory macular hole from multiple etiologies. *Retina*.

[17] D’Souza M. J. J., Chaudhary V., Devenyi R., Kertes P. J., Lam W. C. (2011). Re-operation of idiopathic fullthickness macular holes after initial surgery with internal limiting membrane peel. *British Journal of Ophthalmology*.

[18] Grewal D. S., Mahmoud T. H. (2016). Autologous neurosensory retinal free flap for closure of refractory myopic macular holes. *JAMA Ophthalmology*.

[19] Grewal D. S., Charles S., Parolini B., Kadonosono K., Mahmoud T. H. (2019). Autologous retinal transplant for refractory macular holes: multicenter international collaborative study group. *Ophthalmology*.

[20] Liu P. K., Chang Y. C., Wu W. C. (2018). Management of refractory macular hole with blood and gas-assisted autologous neurosensory retinal free flap transplantation: a case report. *BMC Ophthalmology*.

[21] Chang Y. C., Liu P. K., Kao T. E. (2020). Management of refractory large macular hole with autologous neurosensory retinal free flap transplantation. *Retina*.

